# Impacto de la vacunación primaria con BNT162b2 y una dosis de refuerzo homóloga en los anticuerpos IgA contra SARS-CoV-2 en profesionales sanitarios seronegativos

**DOI:** 10.1515/almed-2022-0051

**Published:** 2022-06-13

**Authors:** Gian Luca Salvagno, Brandon M. Henry, Laura Pighi, Simone De Nitto, Giuseppe Lippi

**Affiliations:** Departamento de Bioquímica Clínica, Universidad de Verona, Verona, Italia; Servicio de Medicina de Laboratorio, Pederzoli Hospital, Verona, Italia; Laboratorio clínico, División de Nefrología e hipertensión, Cincinnati Children’s Hospital Medical Center, Cincinnati, OH, USA

**Keywords:** anticuerpos, COVID-19, inmunoglobulina a, SARS-CoV-2, vacunación

## Abstract

**Objetivos:**

En el presente estudio investigamos la respuesta de los anticuerpos IgA a la vacunación primaria con BNT162b2 y una dosis de refuerzo homóloga, en profesionales sanitarios inicialmente seronegativos.

**Métodos:**

La población de estudio consistió en 69 receptores sanos de la vacuna BNT162b2 (dos dosis), seguida de una única dosis de refuerzo homóloga a los ocho meses. Se extrajeron muestras de sangre a lo largo del estudio, con la primera extracción habiéndose realizado previamente a la primera dosis de la vacuna, y la última hasta un mes después de la dosis de refuerzo. Se midieron los niveles séricos de anticuerpos IgA contra el SARS-CoV-2 con el kit Euroimmun Anti-SARS-CoV-2 *Spike* S1 ELISA IgA.

**Resultados:**

un mes después de la segunda dosis de BNT162b2, se produjo un pico en los niveles de anticuerpos IgA contra el SARS-CoV-2, tras lo cual, fueron disminuyendo paulatinamente los niveles de anticuerpos hasta estabilizarse a los seis meses. La dosis de refuerzo de BNT162b2 (tercera dosis) provocó un segundo pico comparable al observado un mes después de la segunda dosis (p=0,100). Hallamos una correlación muy significativa entre los valores séricos de IgA contra el SARS-CoV-2 antes y después de la dosis de refuerzo (r=0,41; p<0,001), mientras que no se observaron diferencias significativas determinadas por la edad (r=0,10; p=0,416) o el sexo (r=0,04; p=0,729). El índice de receptores seropositivos para IgA contra el SARS-CoV-2 se incrementó del 0% antes de la vacunación, al 80% tras la primera dosis y al 97% tras la segunda dosis de la vacuna, para después disminuir progresivamente al 74% a los tres meses y al 54% a los seis meses, tras lo cual los niveles se estabilizaron. La dosis de refuerzo de BNT162b2 devolvió el índice de seropositividad al 99%.

**Conclusiones:**

Estos resultados fundamentan la propuesta de administrar las dosis de refuerzo de la vacuna tres meses después de la vacunación primaria, con el fin de elevar los niveles de IgA a niveles de protección, especialmente en aquellos grupos de población con mayor riesgo de infección por SARS-CoV-2 o de desarrollar complicaciones.

## Introducción

Aunque algunas de las medidas físicas adoptadas pueden resultar eficaces a la hora de limitar la carga mundial de la enfermedad por coronavirus 2019 (COVID-19) [1], la incesante dilación de tales medidas las hace inviables, por multitud de razones sociales, económicas, e incluso psicológicas [2]. De este modo, la vacunación de la población general contra el síndrome respiratorio agudo severo por coronavirus 2 (SARS-CoV-2) se postula como la estrategia más eficaz a la hora de reducir el riesgo de desarrollar COVID-19 grave (requiriendo hospitalización, ventilación mecánica, cuidados intensivos, etc.), así como para limitar eficazmente la circulación del virus [3]. No obstante, existen varias líneas de evidencia que demuestran notables variaciones en la respuesta individual a las distintas vacunas contra la COVID-19 [4], lo cual se ve reflejado principalmente en la generación heterogénea de anticuerpos neutralizantes contra el SARS-CoV-2, determinada por aspectos individuales como la edad, el sexo, terapias concomitantes y comorbilidades [5]. El estricto seguimiento longitudinal de la inmunidad humoral e identificación de factores predictivos de respuesta a la vacuna son considerados universalmente esenciales a la hora de optimizar la administración de las vacunas [6]. Entre las distintas clases de anticuerpos inducidos por la vacunación, las inmunoglobulinas A (IgA) desempeñan un papel esencial, dado que sus valores séricos están estrechamente relacionados con el desarrollo de inmunidad humoral de las mucosas, que limita considerablemente el riesgo de contagio [7].

En 2021, iniciamos un estudio longitudinal de serovigilancia para realizar una monitorización de seguimiento y analizar la cinética de los niveles séricos de anticuerpos contra el SARS-CoV-2 generados en una cohorte de receptores sanos de la vacuna BNT162b2 [8]. Presentamos un análisis de este ensayo en curso, analizando las variaciones en los anticuerpos IgA contra el SARS-CoV-2 tras la vacunación primaria y recibir una dosis de refuerzo homóloga.

## Materiales y métodos

La población original de estudio consistía en 100 profesionales sanitarios del hospital Pederzoli Hospital (Peschiera del Garda, Italia) inicialmente seronegativos, que recibieron vacunación primaria con BNT162b2 (Pfizer Inc., New York, NY; dos dosis de 30 µg, con 3 semanas de diferencia) contra la COVID-19, seguida de una única dosis de refuerzo (30 µg) ocho meses después de haber completado el ciclo de vacunación primaria. Los análisis moleculares para diagnosticar la infección accidental por SARS-CoV-2 durante el periodo de estudio se realizó en intervalos de 2 o 4 meses, bien con el kit Altona Diagnostics RealStar SARS-CoV-2 RT-PCR (Altona Diagnostics GmbH, Hamburg, Alemania) bien con el análisis Seegene Allplex SARS-CoV-2 (Seegene Inc., Corea del Sur). Se extrajeron muestras de sangre venosa antes de recibir la primera y segunda dosis del ciclo de vacunación primaria, al cabo de 1, 3, y 6 meses, inmediatamente antes de recibir la dosis de refuerzo homóloga de BNT162b2, y al cabo de un mes.

Se midieron los niveles séricos de anticuerpos IgA contra el SARS-CoV-2 *Spike* S1 con el reactivo SARS-CoV-2 ELISA IgA (Euroimmun, Lübeck, Alemania). Esta prueba consiste en un ensayo inmunoabsorbente ligado a enzimas (ELISA), cuyas características técnicas y diagnósticas se hallan ampliamente descritas en la literatura [9, 10]. Brevemente, la imprecisión total del método es <2%, con un valor predictivo negativo y positivo de 99% y 66%, alcanzando una sensibilidad diagnóstica del 100%, frente a una prueba de microneutralización en cultivo celular. Se considera que el resultado de la prueba es positivo cuando el valor sérico de IgA (expresado como razón con respecto al punto de corte) es ≥1,1. Los resultados de los análisis se expresan como medianas y rango intercuartílico (RIC). Se realizaron el test U de Mann-Whitney, el test de Chi cuadrado (con la corrección de Yates), y la correlación de Spearman, empleando Analyse-it (Analyse-it Software Ltd, Leeds, Reino Unido). Todos los sujetos del estudio firmaron un consentimiento informado previamente a recibir la vacuna y a someterse a la monitorización de seguimiento de los niveles de anticuerpos contra el SARS-CoV-2. Este estudio ha sido revisado y aprobado por el Comité de Ética de las provincias de Verona y Rovigo (59COVIDCESC; 3 de noviembre de 2021), y se desarrolló siguiendo los principios de la Declaración de Helsinki y ajustándose a las leyes y regulaciones locales aplicables.

## Resultados

**Figura 1:**
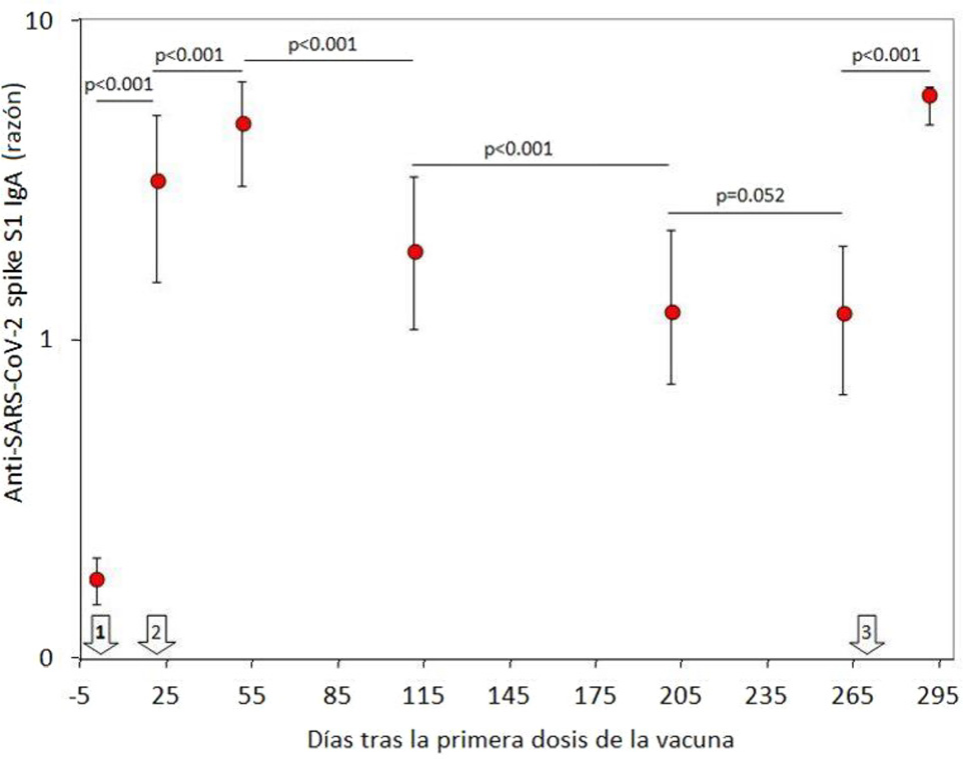
Concentración sérica (mediana y rango intercuartílico) de anticuerpos IgA contra el SARS-CoV-2 *Spike* S1 en una cohorte de profesionales sanitarios vacunados con BNT162b2 y una dosis de refuerzo. Las flechas blancas indican la fecha de las dosis de la vacuna BNT162b2.

**Figura 2:**
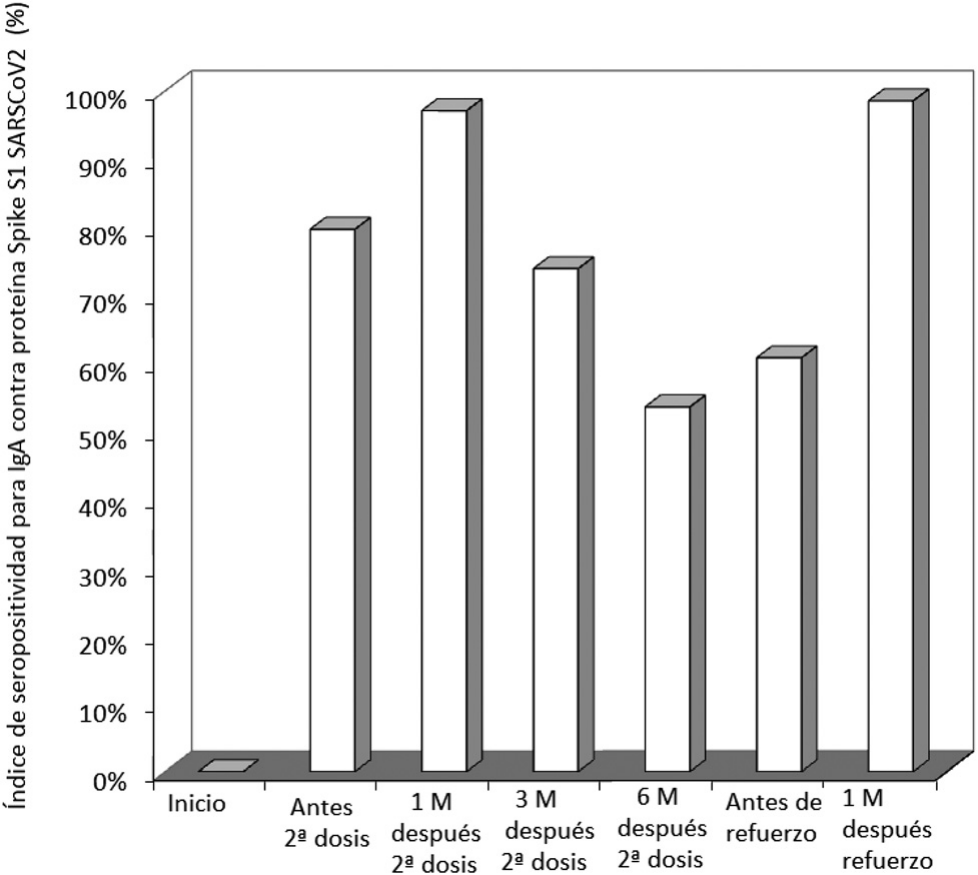
Índice de seropositividad para SARS-CoV-2 *Spike* S1 IgA en una cohorte de profesionales sanitarios vacunados con BNT162b2 y una dosis de refuerzo homóloga. M, meses.

La población final de estudio consistió en 69 profesionales sanitarios inicialmente seronegativos (edad media: 44 años; RIC: 32–52 años; 38 mujeres). Se produjeron 31 pérdidas de seguimiento, al no haber recibido una o más dosis, no haberse podido tomar la muestra de sangre correspondiente, o haber obtenido un resultado positivo en la prueba de ARN de SARS-CoV-2 durante el estudio. La variación de IgA contra la proteína *Spike S1* del SARS-CoV-2 a lo largo del estudio se muestra en la Figura 1. Un mes después de recibir la segunda dosis de BNT162b2, se produjo un pico claramente apreciable de valores séricos de anticuerpos, que disminuyeron progresivamente con el tiempo. Cabe señalar que, ocho meses después de recibir la segunda dosis, dichos niveles se estabilizaron ya que dejaron de diferir significativamente con respecto a los niveles medidos tras la segunda dosis de la vacuna (p=0,100). La administración de la dosis de refuerzo de BNT162b2 (tercera dosis) produjo un segundo pico de IgA contra la proteína *Spike S1* del SARS-CoV-2, que no difería significativamente de los valores observados un mes después de la segunda dosis de la vacuna (Figura 1). Se observó una correlación muy significativa en los valores de IgA contra la proteína *Spike S1* del SARS-CoV-2 antes y después de recibir la dosis de refuerzo (r=0,41; IC95%, entre 0,19 y 0,59; p<0,001) (Figuras 2 y 3), mientras que no se observó ninguna correlación entre los valores de IgA contra la proteína *Spike S1* del SARS-CoV-2 tras la dosis de refuerzo y la edad (r=0,10; IC95%, −0,14 a 0,33; p=0,416) o el sexo (r=0,04; IC95%, −0,20 y 0,28; p=0,729).

**Figura 3:**
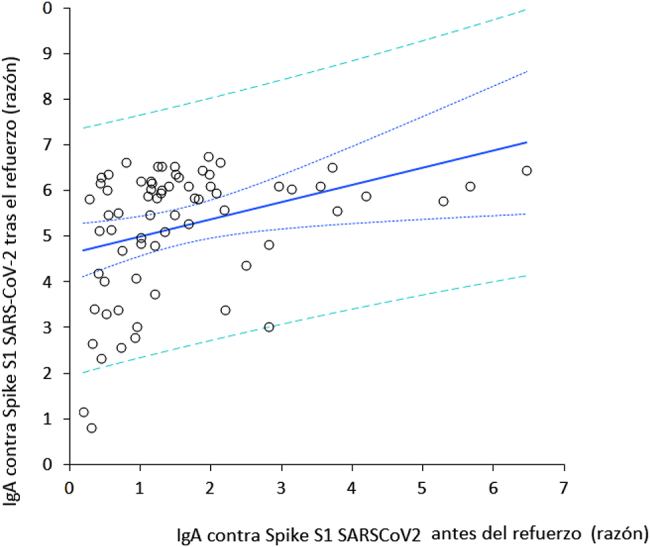
Correlación de Spearman entre la concentraciones séricas de anticuerpos contra la proteína Spike S1 del SARS-CoV-2 antes y después de recibir la dosis de refuerzo de la vacuna BNT162b2.

En la Figura 2 se muestra el índice de receptores de la vacuna con valores de IgA contra la proteína *Spike S1* del SARS-CoV-2 superiores al punto de corte dependiente del método (≥1,1). Tal como se esperaba, el índice se incrementó del 0% previo a la vacunación, al 80% y el 97% tras recibir la primera y segunda dosis de la vacuna, respectivamente. Paralelamente a los niveles séricos, el índice de sujetos seropositivos también disminuyó con el tiempo, pasando del 74% a los tres meses, al 54% a los seis meses, tras lo cual los valores se estabilizaron en alrededor del 60%. La administración de la dosis de refuerzo de BNT162b2 (tercera dosis) elevó el índice de seropositividad para IgA contra el SARS-CoV-2 IgA hasta el 99%.

## Discusión

En el presente estudio, ofrecemos un amplio análisis de la cinética de los anticuerpos IgA contra el SARS-CoV-2 tras la vacunación primaria con BNT162b2 y la administración de una dosis de refuerzo homóloga, en profesionales sanitarios sanos. Se han realizado varias observaciones importantes.

En primer lugar, demostramos que la variación en los niveles de anticuerpos IgA contra el SARS-CoV-2 reflejan parcialmente los niveles séricos de IgG contra la proteína trimérica *Spike* del SARS-CoV-2 [11]. No obstante, a diferencia de los anticuerpos IgG, observamos que los niveles séricos de IgA parecen mostrar una disminución menor con el tiempo, permaneciendo estables a partir de los seis y ocho meses, tras completar el ciclo de vacunación primaria. Del mismo modo, el índice de seropositividad también fue disminuyendo con el tiempo, para estabilizarse relativamente (en alrededor del 60%) a los ocho meses de la vacunación primaria. Esto parece contradecir los datos obtenidos al medir los niveles de anticuerpos IgG contra la proteína trimérica *Spike* del SARS-CoV-2, cuyos índices de protección siguen disminuyendo con el tiempo. Aun así, el relativamente elevado número de sujetos seronegativos para IgA del SARS-CoV-2 ya identificado a los seis meses de la vacunación primaria hace necesaria la administración de una dosis de refuerzo, especialmente en los grupos de población más vulnerables, esto es, los que pueden presentar una evolución desfavorable de la enfermedad, así como en aquellos que vuelven a ser seronegativos con el tiempo.

Los resultados también demuestran que los niveles séricos de IgA contra la proteína *Spike* S1 del SARS-CoV-2 antes de la dosis de refuerzo eran predictores de los valores tras recibir dicha dosis, no habiéndose observado relación entre dichos valores y la edad o el sexo. Esto sugiere que los sujetos que muestran una menor disminución de los valores de IgA contra el SARS-CoV-2 pueden ser los que desarrollen una mejor respuesta a la dosis de refuerzo homóloga, lo cual es importante a la hora de optimizar las vacunas contra la COVID-19. Debido a las dificultades a la hora de garantizar la vacunación universal contra la COVID-19 [12], especialmente en los países subdesarrollados en los que existe escaso acceso a las vacunas, personalizar la vacunación (en términos de dosis y pauta temporal) permitiría ahorrar recursos tanto humanos como económicos. El estudio también revela una diferencia con la tendencia de los anticuerpos IgG contra la proteína trimética *Spike* del SARS-CoV-2, cuyo segundo pico se produce al mes de recibir la dosis de refuerzo. Así, estos valores fueron tres veces superiores que los observados tras el primer pico [11], mientras que los dos picos de anticuerpos IgA contra el SARS-CoV-2 fueron similares. Así mismo, teniendo en cuenta la importante función que desempeñan los anticuerpos séricos IgA en la protección del organismo contra el SARS-CoV-2, así como que estos reflejan con precisión el desarrollo de inmunidad humoral en las mucosas [7], creemos que, de forma complementaria a los estudios de seroprevalencia, se pueden medir los niveles de estos anticuerpos, para evaluar la respuesta humoral a la vacuna y a las dosis de refuerzo contra la COVID-19.

En conclusión, habida cuenta de la estrecha correlación existente entre los niveles de anticuerpos IgA neutralizantes contra el SARS-CoV-2 séricos y secretores [13], los resultados de este estudio de seroprevalencia respaldan la propuesta de administrar una dosis de refuerzo tres meses después de la vacunación primaria, con el fin de recuperar los niveles de protección de los IgA, especialmente en los sujetos con mayor riesgo de contraer la infección por SARS-CoV-2 o de desarrollar complicaciones [14].
